# A Manual of Medicine

**Published:** 1903-09

**Authors:** 


**A Manual of Medicine.** By THOMAS KIRKPATRICK MONRO, M.A., M.D. Pp. xx., 901. London: Baillière, Tindall, and Cox. 1903.

Some books are too large, some are too small, this one is considered to be the right size for the average student of medicine and junior practitioners. It does not profess to be exhaustive, but it gives just what the student must know and should know before he goes to more voluminous monographs on special subjects.

The first section, on Specific Infectious Diseases, includes Rheumatic Fever, Tuberculosis, Lobar Pneumonia, Syphilis, and various other less common maladies, such as Dengue, Frambœsia, and other tropical diseases. The description of these is terse, but comprehensive and reliable. A compression of all the important points of these forty-one different maladies into the space of 191 pages is worthy of all commendation, and we have failed to find any serious omission or inaccuracy.

Section II., Constitutional Diseases, includes Chronic Rheumatism and Rheumatoid Arthritis, which are here associated with gout rather than with acute rheumatism. We think this is wrong, and we question if patients with rheumatic myalgia would approve of the insertion of long needles into the painful muscles as recommended by the author. A section on Diseases of the Skin, one on Animal Parasites, and a very complete index, conclude a volume which is eminently fitted for the purpose intended by the author, who is already well known to the medical reader by his *Monograph on Raynaud's Disease* and by his *History of the Chronic Degenerative Diseases of the Central Nervous System.*

